# Effects of MnTBAP on Porcine Semen Cryopreservation and Capacitation

**DOI:** 10.3390/antiox13060672

**Published:** 2024-05-30

**Authors:** Eunji Kim, Il-Jeoung Yu, Joohyeong Lee, Yubyeol Jeon

**Affiliations:** 1 Department of Theriogenology and Reproductive Biotechnology, College of Veterinary Medicine, Jeonbuk National University, Iksan 54596, Republic of Korea; keejj3@jbnu.ac.kr (E.K.); iyu@jbnu.ac.kr (I.-J.Y.); 2 Department of Companion Animal Industry, Semyung University, Jecheon 27136, Republic of Korea

**Keywords:** MnTBAP, porcine semen, cryopreservation, antioxidant, capacitation

## Abstract

Antioxidants protect cellular function and structure by neutralizing the oxidative stress caused by increased reactive oxygen species (ROS) during sperm freezing. Studies on cryopreservation using various antioxidants have demonstrated encouraging results. Many studies have used antioxidants to increase the efficiency of sperm freezing and to improve the success rate of artificial insemination and pregnancy. Manganese (III) tetrakis (4-benzoic acid) porphyrin chloride (MnTBAP) is a newly synthesized antioxidant with positive effects on sperm morphology and capacitation in humans, rams, and stallions. In this study, porcine semen was treated with 0, 50, 100, and 150 μM of MnTBAP based on a Tris–egg-yolk extender and frozen to determine whether MnTBAP can assist the status of sperm during cryopreservation. First, motility was assessed using the computer-assisted sperm analysis (CASA) system, with the 100 μM treatment group showing the highest motile rate (66.8%) compared with that of the other groups (control, 51.1%; 50 μM and 150 μM, 59.6%); therefore, the remaining analyses were conducted comparing the two groups (control vs. 100 μM group; *p* < 0.01). Second, fluorescence staining was applied to examine the control and 100 μM groups using fluorescence microscopy. The viability (41.7% vs. 62.4%) and the acrosome integrity (77.9% vs. 86.4%) differed significantly (*p* < 0.05). In addition, the mitochondrial membrane potential (MMP) was 46.5% vs. 51.9%; the fragmentation rate, estimated using the Sperm-sus-Halomax kit, was 63.4% vs. 57.4%; and the detected caspase activity was 30.1% vs. 22.9%. These tended to be higher in the treated group but did not differ significantly. Third, measurements using FACSLyric revealed that the 100 μM treatment group exhibited a state of elevated normal lipid arrangement within the plasma membrane and diminished levels of apoptosis and ROS (*p* < 0.01). We assessed the expression of genes relevant to antioxidant effectiveness using real-time RT-qPCR. Our findings indicated significant alterations in the expression levels of various mRNA species, with the exception of NOX5 (*p* < 0.05). Finally, the straws were dissolved and used to treat matured denuded oocytes to investigate the effect on fertilization and embryo development in vitro. The cleavage rate was (77.6% vs. 84.1%), and the blastocyst rate was 9.7% vs. 11.4% (*p* < 0.05). In conclusion, these results suggest that MnTBAP positively affected sperm freeze–thawing, improving the fertilization capacity, and leading to increased embryo development.

## 1. Introduction

Germplasm cryopreservation is a pivotal technique for managing infectious disease transmission, conserving genetic diversity, and aiding population management in various species [1]. Based on Spallanzani’s initial efforts in human sperm cryopreservation, progress has been made in methodological approaches for various species such as cattle, sheep, pigs, and horses. These advancements have resulted in successful outcomes, including the birth of offspring using freeze–thawed sperm in artificial insemination [2,3].

Subsequently, glycerol was observed to function as a cryoprotective agent (CPA), effectively enhancing the efficiency of freezing semen, tissues, blood cells, and bacteria [4,5]. The extent of the structural impairment of membrane phospholipids depends on both the temperature and composition of the membrane phospholipids. Spermatozoa undergo significant stress due to rearrangement, destabilization, and calcium influx of the sperm plasma membrane during transitions in temperature, such as from body temperature to cooling and freezing [6,7]. Pigs contain a higher concentration of polyunsaturated fatty acids than other species, which is associated with membrane fluidity and flexibility; however, this characteristic increases the risk of cryodamage during freezing [8]. Furthermore, excessive fatty acid content leads to the generation of reactive oxygen species (ROS) in the mitochondria, resulting in DNA damage and functional deprivation [9]. 

ROS, including oxygen metabolites such as H_2_O_2_, O_2_^•−^, and ^•^OH are generated during the regular metabolic processes of aerobic cells and exhibit high reactivity with carbohydrates, lipids, proteins, and nucleic acids [10]. A low level of ROS production by sperm is crucial as it regulates protein tyrosine phosphorylation pathways mediated by PKA and ERK, thereby impacting capacitation or serving as cellular messengers [11]. However, since the initial discovery of the detrimental effects of ROS on sperm in 1943, numerous studies have highlighted the ability of ROS to disrupt the structural, physiological, and functional characteristics of sperm. Specifically, ROS can induce damage to the mitochondrial DNA (mtDNA), which is closely linked to motility, resulting in genomic, proteomic, lipidomic, and epigenomic alterations associated with impaired sperm function and infertility. Furthermore, ROS-induced cellular damage activates caspase activity, leading to apoptosis and DNA fragmentation such as in chromatin condensation and plasma membrane blebbing. Spermatozoa with DNA damage exhibit impaired capacitation and abnormal developmental progression [12,13,14,15]. 

Although seminal plasma contains enzymatic and non-enzymatic antioxidants, they become present in trace amounts after washing and dilution with cryo-media, resulting in an imbalance between ROS and natural scavenger activity after freezing. This imbalance decreases the cleavage rate, embryo development, and blastocyst formation after fertilization [16]. Despite ongoing efforts to mitigate these issues through the application of exogenous antioxidants, artificial insemination (AI) requires large semen volumes and typically results in lower conception rates than natural fertilization using fresh semen. Consequently, it is imperative to explore novel antioxidants and delve deeper into their intracellular mechanisms of action to address this challenge effectively. Resveratrol (RSV), quercetin, catalase (CAT), melatonin, and cysteine are antioxidants that have demonstrated efficacy in various species [17,18,19,20,21].

Manganese (III) tetrakis (4-benzoic acid) porphyrin chloride (MnTBAP), a newly synthesized member of the metalloporphyrin family of antioxidants, has scavenging abilities similar to those of superoxide dismutase (SOD) [22]. It functions analogously to SOD in modulating the redox signaling pathway and has demonstrated beneficial effects on the lung, immune system, and cardiac function [23,24,25]. Treatment with media during freezing or thawing exerts a favorable influence on motility, viability, capacitation status, and blastocyst formation. MnTBAP contributes to the reduction of ROS levels in the semen of various species, including humans, stallions, goats, and sheep [26,27,28,29]. 

We investigated the effective concentration of MnTAP as an antioxidant by adding various concentrations of MnTBAP to cryoprotectants when freezing boar semen. Subsequently, we evaluated the viability, acrosome integrity, DNA damage, caspase activity, and plasma membrane lipid disorder at the most effective concentration determined based on sperm motility, which is essential for sperm experiments. We measured apoptosis and ROS levels to evaluate the antioxidative responses. In addition, we assessed embryo developmental rates via in vitro fertilization to understand the impact of cryopreserved semen.

## 2. Materials and Methods

### 2.1. Experimental Design

All reagents and chemicals used in this study were purchased from Sigma-Aldrich (St. Louis, MO, USA) unless otherwise indicated. The aim of this study was to assess the effectiveness of MnTBAP supplementation as an antioxidant during the cryopreservation of porcine semen. We utilized 100 ejaculates (motility > 90%) from different boars in this study. Initially, parameters such as motility, viability, acrosome integrity, and plasma membrane lipid arrangement were assessed to evaluate structural alterations and changes in spermatozoa following exposure to cryoprotective agents and low temperatures during freezing. Subsequently, the mitochondrial membrane potential, caspase activity, and apoptosis levels were measured to examine the physiological repercussions of changes in osmolality, pH, and reactive oxygen species production due to sperm metabolism after freezing and thawing. Finally, in vitro fertilization assays were conducted to confirm sperm capacitation capability, embryo development was monitored, and RT-qPCR was performed to ascertain antioxidant-related gene expression patterns.

### 2.2. Preparation of Cryoprotectants

The extender was based on Beltsville F5 (BF5) [30] and comprised 12 g/L Tes-N-Tris (hydroxymethyl) methyl 2 aminoethane sulfonic acid, 2 g/L Tris (hydroxymethyl) amino-methane, 32 g/L D (+) glucose, 1.5% (*v*:*v*) Orvus ES Paste (Minitube, Tiefenbach, Germany), 20% (*v*:*v*) egg yolk, and 0.02 g/L gentamycin sulfate. This was used as Extender 1, while Extender 2 contained 4% (*v*:*v*) glycerol. MnTBAP was used in each extender at concentrations of 0, 50, 100, and 150 μM. The optimal concentration of MnTBAP was determined based on the motility results using several parameters. All parameters were measured at least three times.

### 2.3. Procedure of Cryopreservation and Thawing

Ejaculated semen from Duroc boars was washed, diluted with Beltsville Thawing Solution (BTS), and stored at 17 °C in semen packs obtained from the Korea Pig Genetic (KPG, Gimje, Republic of Korea) Center. The packs were transported to the laboratory within 1 h of processing. After dispensing, the semen was temperature-stabilized at 17 °C for at least 2 h before use. Only semen packs purchased on the same day were used to maintain consistent conditions for each freezing cycle. Samples with motility exceeding 90% were selected for cryopreservation. The semen was centrifuged at 1200 rpm for 7 min at 17 °C. The supernatant was removed, and the pellets were resuspended in BTS solution containing 37.0 g/L anhydrous glucose, 6.0 g/L dihydrous sodium citrate, 1.25 g/L sodium bicarbonate, 12.5 g/L Na-EDTA, 0.75 g/L potassium chloride, 0.6 g/L sodium penicillin G, and 1.0 g/L streptomycin [31]. The pellet was obtained by centrifugation at 1200 rpm and 17 °C for 7 min, and the supernatant was removed. An aliquot of the pellet was diluted with 1 N HCl and counted in a cell-counting chamber (Marienfeld, Lauda-Königshofen, Germany). The sperm was mixed with Extender 1 to a concentration of 2 × 10^8^ cells/mL and cooled at 4 °C for 1 h. Then, an equal volume of Extender 2 was added to the mixture, loaded in a 0.5 mL straw (Minitube, Tiefenbach, Germany), and sealed. The final sperm concentration in each straw was 1 × 10^8^ cells/mL. The straws underwent a second cooling process at 4 °C for 25 min to stabilize. Liquid nitrogen (LN_2_) was added up to 8 cm from the bottom of the Styrofoam box, and the straw was placed on a rack located 4 cm above the LN_2_ surface. The straws were then exposed to the LN_2_ vapor for 20 min and frozen. The frozen straw was transferred to a goblet and stored in LN_2_ until further analysis. The straws were thawed by immersion in a 38 °C water bath for 30 s immediately before being utilized in the study.

### 2.4. Motility

Upon thawing, the sample was maintained at 38 °C, and 2 μL was taken and placed into a standard count 20 μM 8-chamber slide (Leja, Nieuw-Vennep, The Netherlands) for observation under a microscope (Nikon E-200, Minato-ku, Tokyo, Japan). The samples (control, 50 μM, 100 μM, and 150 μM MnTBAP) were then analyzed using a computer-assisted sperm analysis (CASA) system, which categorized sperm motility into total motile (TM), progressive motile (PM), rapid progressive motile (RPM), medium progressive motile (MPM), non-progressive (NP), and immotile (IM) based on the movement patterns observed.

### 2.5. Viability

Viability was measured by staining with the LIVE/DEAD Sperm Viability Kit (ThermoFisher, Waltham, MA, USA) in accordance with the manufacturer’s instructions. Briefly, thawed semen was diluted with 1× Dulbecco’s phosphate-buffered saline (DPBS, Gibco, Scotland, UK) (1:4, *v*:*v*), and 50 μL was transferred to a 1.5 mL tube. Subsequently, 5 μL of 0.2 mM SYBR-14 was added and incubated for 5 min in the dark at room temperature (RT; 25 °C). Thereafter, 5 μL of 0.24 mM propidium iodide (PI) was added and the mixture was incubated for 5 min under the same conditions. Then, a smear was made using 10 μL aliquots. At least 200 sperm were counted in various fields under a fluorescence microscope (Axio, Carl Zeiss, Goettingen, Germany) after drying. SYBR-14 emits a strong green light using a 488/516 nm filter, indicating living sperm. PI emits a strong red light with a 535/617 nm filter, indicating dead sperm [32].

### 2.6. Acrosome Integrity

We used the pisum sativum agglutinin (PSA) and fluorescein isothiocyanate (FITC) method [33]. In short, the sample was prepared by dilution, as mentioned above, and a smear was created using 20 μL of the sample. After drying, methanol was dropped onto the slide, fixed, and dried again. The dye mixture (100 mg/mL in PBS) was placed on a slide and attached to parafilm (Bemis, Chicago, IL, USA) for even staining. The smear was incubated in the dark for 20 min at RT, and the parafilm was eliminated by immersion in distilled water. Finally, the slides were dried and analyzed under a fluorescence microscope (Axio, Carl Zeiss, Goettingen, Germany) by counting more than 200 spermatozoa. An intact acrosome, with PSA equally distributed across its surface, demonstrated a strong green fluorescence. Conversely, the presence of a faint green fluorescent band near the equator or the absence of fluorescence indicated damage to the acrosome.

### 2.7. Mitochondrial Membrane Potential (MMP)

We examined MMP using 0.01 mg/mL rhodamine 123 (Molecular Probes, Eugene, OR, USA) and 2.4 mM PI [34]. A 250 μL thawed sample was prepared, and 5 μL of rhodamine and 5 μL of PI were added sequentially and mixed, and then the mixture was incubated in the dark at RT for 15 min. A smear was made using 10 μL of the stained sample. Over 200 spermatozoa were observed under a fluorescence microscope (Axio; Carl Zeiss, Goettingen, Germany). A normal MMP exhibits strong green fluorescence exclusively in the midsection of the sperm, whereas a low MMP is distinguished by strong red fluorescence in the head of the sperm and weak green light in the midsection. 

### 2.8. DNA Fragmentation

DNA damage was measured by following the instructions of the Sperm-sus-HaloMax kit (Halotech DNA, Madrid, Spain) and of the modified protocol from [35,36]. First, the agarose gel tube was melted at 95 °C for 5 min. Then, the gel was warmed at 38 °C for an additional 5 min, and 25 μL of the thawed sample was mixed with 50 μL of the gel. Subsequently, 2 μL was dispensed into the specially coated zone on the slide and covered with a cover slip. The slide was cooled at 4 °C for 5 min for the gel to solidify. The slide and cover were gently separated, maintaining the gel shape, and the lysis buffer was placed horizontally on the slide for 5 min. The buffer was washed with distilled water and soaked in purified water for 5 min. Thereafter, the slides were sequentially treated with 70% and 100% ethanol for 2 min each. Before staining, the slides were dried at RT. Staining was performed using the Diff-Quik stain (Sysmex, Kobe, Japan). Diff-Quik was applied by dipping the slides five to ten times in Diff Quik solutions I and II, rinsing with distilled water, and drying to prepare a sample for examination. Two hundred spermatozoa were scored under a 100 × objective lens (Leica DM2500, Wetzlar, Germany) using immersion oil (Leica Type N, Wetzlar, Germany). We divided the samples into two groups: (1) large halo: DNA fragmented with nucleoid diffusion of chromatin spots, and (2) non-halo: sperm nuclei without DNA fragmentation.

### 2.9. Caspase Activity

Caspase activity was analyzed using a CaspGLOW Red Active Staining Kit and a Fluorescein Active Caspase Staining Kit (Bio-Vision, Milpitas, CA, USA) following the manufacturer’s instructions and as previously described [37]. Initially, 0.5 mL of thawed semen was diluted with 3.2 mL of DPBS, and 300 μL of the mixture was transferred to a new tube. Subsequently, 1 μL of sulforhodamine was added, and the sample was incubated at 37 °C for 20 min in the dark. The tubes were centrifuged at 860× *g* for 5 min. After discarding the supernatant, the pellet was resuspended in 500 μL of DPBS, and 10 μL of the sample was placed on a slide and covered with a coverslip. Fluorescence microscopy (Axio, Carl Zeiss, Goettingen, Germany) was used to analyze the cells. Positive cells were identified by vigorous red fluorescence in the midsection and tail, whereas negative cells exhibited weak or no fluorescence.

### 2.10. Plasma Membrane Lipid Disorder

The procedures were performed according to the protocol outlined previously [38]. Merocyanine 540 (M540) and YoPro-1 were used to assess the plasma membrane lipid organization in spermatozoa. In summary, thawed samples were centrifuged at 300× *g* for 3 min, and the supernatant was carefully removed. Then, 500 μL of DPBS was mixed carefully, followed by centrifugation under the same conditions to remove the supernatant. The pellet was resuspended in another 500 μL DPBS and stained with 75 nM YoPro-1 and 6 μM M540. The mixture was incubated at 38 °C for 15 min in the dark before measurements were taken. M540 exhibited a high affinity for the membrane, indicating increased lipid disorder, and emitted strong fluorescence when observed through the 572 ± 28 nm bandpass filter. In contrast, YoPro-1, correlated with sperm viability, is a non-permeable stain that enters membrane-compromised sperm and was detected using the 530 ± 30 nm bandpass filter from the FACSLyric Flow Cytometry system (BDbiosciences, Eusins, Switzerland). The detection section was divided into three groups: (1) M540+/YoPro-1+ and M540−/YoPro-1+: dead spermatozoa, (2) M540+/YoPro-1−: live spermatozoa with high lipid disorder, and (3) M540−/YoPro-1−: live spermatozoa with low lipid disorder.

### 2.11. Apoptosis

The FITC Annexin V apoptosis detection kit I (BD Biosciences, San Diego, CA, USA) was used in conjunction with PI to measure apoptosis caused by the freeze–thaw procedure. In short, after thawing the semen straws of both the control and 100 μM MnTBAP-treated groups and preparing the samples, they were centrifuged at 312× *g* for 3 min to form pellets. The supernatant was then discarded, and the pellets were resuspended in 500 μL DPBS. After further centrifugation at 300× *g* for 3 min, the liquid, excluding the pellet, was removed. Subsequently, 500 μL DPBS was added to dissolve the pellet, and then 980 μL of 1× Annexin V binding buffer (0.1 M HEPES in NaOH (pH 7.4), 1.4 M NaCl, and 25 mM CaCl_2_) (10× buffer: distilled water = 1:9 (*v*:*v*)) and 20 μL of sperm suspension were mixed. Next, 100 μL of the mixture was transferred to a FACS tube (Falcon, Tamaulipas, Mexico). Sequentially, 5 μL of Annexin stain and 5 μL of PI were added and incubated under dark conditions at room temperature for 15 min. Before measurement using the FACSLyric Flow Cytometry system (BDbiosciences, Eusins, Switzerland), 400 μL of 1× Annexin buffer was added, and all evaluations were conducted within 1 h after thawing. The analysis was divided into four distinct groups as follows: (1) FITC−/PI−: viable and non-apoptotic; (2) FITC+/PI−: viable, phosphatidylserine (PS) translocated, and pro-apoptotic; (3) FITC+/PI+: dead and PS translocated; and (4) FITC−/PI+: late necrotic sperm. The apoptosis index was indicated as the ratio of (FITC+/PI−)/total PI−.

### 2.12. Reactive Oxygen Species (ROS)

Following sample thawing and preparation, centrifugation was performed at 312× *g* for 3 min. After discarding the supernatant, the pellet was resuspended in 500 μL of DPBS and subjected to centrifugation under identical conditions. After discarding the supernatant once again and completing the extender removal process, the pellet was fully resuspended in 500 μL of DPBS. Subsequently, 50 μL of the sample and 950 μL of DPBS were transferred to a 5 mL FACS tube (Falcon, Tamaulipas, Mexico). Then, 1 μL of 20 mM 2′,7′-dichlorodihydrofluoresceindiacetate (H_2_DCFDA DCF, Invitrogen, Carlsbad, CA, USA) and 1 μL of 2.4 mM PI were added, followed by incubation under dark conditions for 1 h. Fluorescence measurements were performed within 1 h using the FACSLyric Flow Cytometry system (BDbiosciences, Eusins, Switzerland) with a 530/30 nm bandpass filter for DCF and a 5885/42 nm bandpass filter for PI. We divided the analysis into four distinct groups as follows: (1) DCF−/PI−: alive and low H_2_O_2_; (2) DCF+/PI−: viable and high H_2_O_2_; (3) DCF+/PI+: dead and high H_2_O_2_; and (4) DCF−/PI+: dead and low H_2_O_2_. The ROS index represents the proportion of viable sperm with low H_2_O_2_ levels among the total number of viable sperm.

### 2.13. Gene Expression

Total RNA for gene expression analysis was extracted from 2 groups (control and 100 μM MnTBAP) using TRIzol reagent (Invitrogen, Carlsbad CA, USA) following the manufacturer’s method and as previously described [37]. Briefly, spermatozoa were thawed at 37 °C for 30 s, washed twice with DPBS by centrifugation at 160× *g* for 2 min, and treated with TRIzol reagent. After mixing and incubating for 5 min at RT, chloroform was added, and the mixture was incubated for another 5 min. The mixtures were centrifuged at 1600× *g* at 4 °C for 10 min. Subsequently, 100 μL of the upper aqueous phase was mixed with 100 μL of isopropanol (*v*:*v*) and 7 μL of glycogen (Biosolution, Suwon-si, Republic of Korea). The samples were incubated overnight at 4 °C to ensure sufficient RNA precipitation. The samples were centrifuged at 13,000 rpm for 15 min at 4 °C, the supernatant discarded, and the RNA pellet washed with 70% ethanol. Then, the RNA pellet was dissolved in DEPC water and the concentration measured using a microplate spectrophotometer (Epoch Microplate spectrophotometer, Agilent, Santa Clara, CA, USA). Reverse transcription-quantitative PCR (RT-qPCR) was conducted using the primers listed in Table 1 and the One-Step TB Green Prime Script RT-PCR Kit II (Takara, Bio USA, Inc., Mountain View, CA, USA) on the ABI 7500 real-time PCR system (Applied Biosystems, Beverly, MA, USA). The PCR settings consisted of (1) an RT step (42 °C, 5 min and 95 °C, 10 s); (2) 40 PCR cycles (95 °C, 34 s and 54 °C, 34 s); and (3) a dissociation step. The following mRNAs were observed: *BAX*, Bcl-2-associated X protein; *Bcl-2*, Bcl-2 apoptosis regulator; *ROMO1*, reactive oxygen species modulator 1; *SMOX*, spermine oxidase; *NOX5*, NADPH oxidase 5; *SMCP*, sperm mitochondria-associated cysteine-rich protein; *SOD1*, superoxide dismutase 1; and *catalase*. The *GAPDH* mRNA was used for normalization. The gene quantitative expression levels were determined using the equation nR = 2^−(∆Ct sample − ∆Ct control)^. 

### 2.14. In Vitro Maturation (IVM)

The media used for IVM were based on TCM-199 (m199, Gibco, NY, USA) and had the following compositions: (1) m199 (+): m199 with 10% porcine follicular fluid (pFF; *v*:*v*), 0.6 mM cysteine, 0.91 mM sodium pyruvate, 10 ng/mL epidermal growth factor (EGF), 75 µg/mL kanamycin, and 1 µg/mL insulin; and (2) m199 (++): m199 (+) with 10 IU/mL equine chronic gonadotropin (eCG; DAESUNG, Gyeonggi, Republic of Korea) and 10 IU/mL hCG (DAESUNG, Gyeonggi, Republic of Korea). Ovaries were retrieved from the slaughterhouse and transported to the laboratory within 1 h, where they were stored in 0.9% NaCl solution at 38 °C. Follicles with diameters of 3–6 mm were aspirated using a 15 mL syringe and an 18 G needle, and the oocytes were collected in a 15 mL tube. The supernatant was discarded after a 10 min incubation in a 38 °C water bath to settle the cells and debris. HEPES-buffered Tyrode’s medium (TLH) containing 0.05% polyvinyl alcohol (PVA) (TLH-PVA) was added for the primary wash, followed by a secondary wash with TLH-PVA for 5 min. After removing the supernatant, oocytes covered with at least three layers of cumulus cells with intact oolemma, a normal spherical shape, and dark granular cytoplasmic morphology were selected using a mouth pipette. The selected oocytes were washed twice with m199 (+) medium, and then approximately 50 oocytes were transferred into 500 μL m199 (++) medium and cultured for 22 h at 38 °C in an incubator under 5% CO_2_ and humidified atmosphere conditions. Subsequently, oocytes were washed once with m199 (+) medium, followed by additional culturing under the same conditions in m199 (+) media for 18–20 h. 

### 2.15. In Vitro Fertilization (IVF)

Mature oocytes were denuded of cumulus cells by pipetting into TLH containing 0.1% hyaluronidase. Oocytes at the MII stage were selected based on confirmation of the polar body under a microscope (Leica, MSV269, Wetzlar, Germany) and placed in drops of modified Tris-buffered medium (mTBM) covered with heavy mineral oil (IrvineScientific, Fujifilm, ST. Santa Ana, CA, USA), with 15 oocytes per 40 μL mTBM drop. For sperm preparation, semen straws were thawed at 38 °C for 30 s and diluted with DPBS. Sperm pellets were obtained by centrifugation at 2000 rpm for 2 min, and after removing the supernatant, the pellet was resuspended in DPBS and centrifuged again under the same conditions. Then, 100 μL mTBM was added to the pellet, and 10 μL of the sperm suspension was mixed with 190 μL of 1 N HCl to immobilize the sperm for counting. The sperm count was determined using a cell counter, and the dilution rate depended on the total motility of the CASA system. We designated this concentration as 2 × 10^6^ motile sperm/mL, and 5 µL of the prepared sperm suspension was spread evenly onto a drop of mTBM containing oocytes along the edge. The fertilization process was conducted for 5 h at 38 °C and 5% CO_2_ in an incubator in the dark.

### 2.16. In Vitro Culture (IVC)

After 5 h of fertilization, sperm denudation was performed in TLH, and each batch of 10 presumptive zygotes was cultured in 25 μL porcine zygote medium 3 (PZM-3) at 5% CO_2_, 5% O_2_, and 38 °C in an incubator. The day of fertilization was designated as day 0. The cleavage rate was evaluated on day 2, and blastocyst formation was assessed on day 7 to analyze embryo development. Data were collected from a minimum of three experimental trials. 

### 2.17. Statistical Analysis

All data are represented as means ± S.E.M. Differences between two groups were assessed using the Student *t*-test, while comparisons including more than two groups were analyzed using one-way ANOVA. GraphPad Prism version 9.3.1 was used for statistical analysis. Asterisks represent significant differences between the control and treatment groups (* *p* < 0.05, ** *p* < 0.01, and *** *p* < 0.001).

## 3. Results

### 3.1. Motility

**Figure 1:**
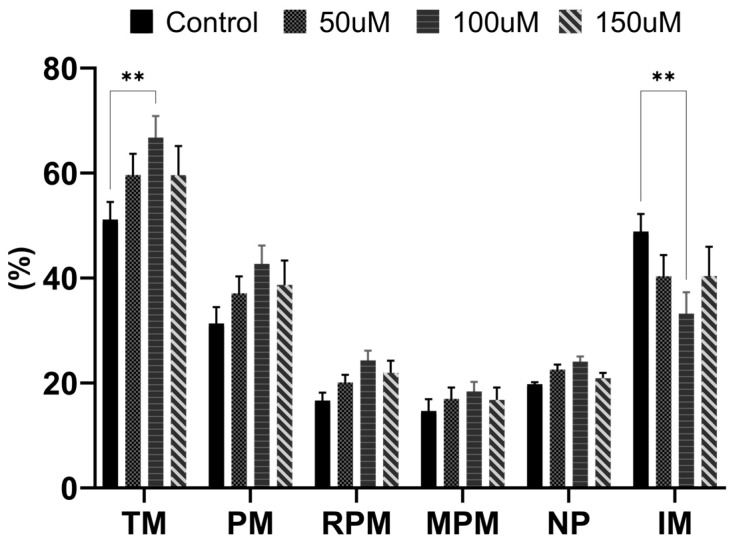
The effects of different manganese (III) tetrakis (4-benzoic acid) porphyrin chloride (MnTBAP) concentrations (0, 50, 100, and 150 μM) on the motility of cryopreserved porcine sperm. TM: total motility; PM: progressive motile; RPM: rapid progressive motile; MPM: medium progressive motile; NP: non-progressive; IM: immotile. Data are presented as means ± S.E.M. The asterisk indicates a significant difference between the two groups (** *p* < 0.01).

We conducted a comparative analysis of cell motility between the control and MnTBAP-treated groups at different concentrations. Our findings revealed that the treated groups exhibited enhanced motility compared with that of the control group. Specifically, the group treated with 100 μM MnTBAP displayed significantly elevated TM and reduced IM, indicating that this concentration was the most efficacious in preserving sperm movements (Figure 1) (*p* < 0.01). Based on the motility results, in this study, subsequent parameters were analyzed by comparing the control and the 100 μM groups.

### 3.2. Viability

**Figure 2:**
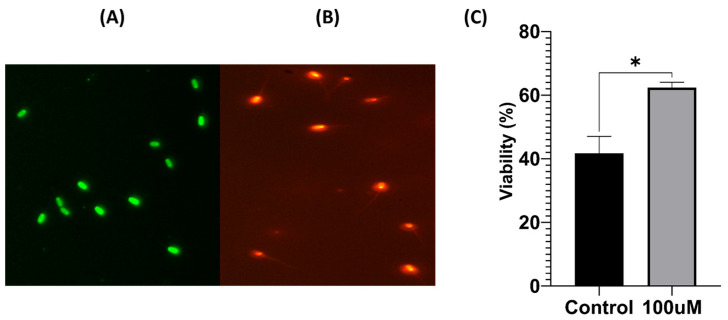
MnTBAP increased the viability of pig sperm after freezing and thawing. (**A**) The live sperm was stained with SYBR-14, and the sperm head showed a strong green fluorescence. (**B**) The dead sperm was stained with propidium iodide (PI), and the sperm head showed strong red fluorescence. (**C**) The percentage of live sperm among the total counted sperm. Data are displayed as means ± S.E.M. The asterisk indicates a significant difference between the two groups (* *p* < 0.05).

The sperm viability results are shown in Figure 2. Live sperm stained with SYBR-14 are illustrated in (A), while (B) shows dead sperm stained with PI. The total count of live sperm within the observed field is presented in Figure 2C. This was approximately 1.5 times higher in the treated group than in the untreated group. Analysis of the ratio of dead to live sperm suggested a favorable effect of 100 μM MnTBAP on sperm viability during the freezing–thawing process (* *p* < 0.05).

### 3.3. Acrosome Integrity

**Figure 3:**
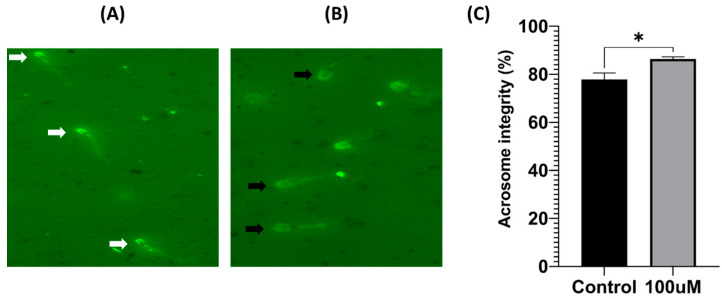
Treatment with 100 μM of MnTBAP improved the acrosome integrity of pig sperm cryopreservation. (**A**) White arrows indicate the normal acrosome structure after freezing and thawing. (**B**) Black arrows indicate damaged acrosomes. (**C**) The ratio of acrosome integrity in stained sperm. Data are indicated as means ± S.E.M. The asterisk indicates a significant difference between the two groups (* *p* < 0.05).

A strong fluorescence was observed in the acrosome region in (A) of Figure 3, while (B) depicts weak or non-fluorescent sperm. A comparison between the acrosome integrity of the control and the 100 μM treatment groups suggested that the treatment protected the acrosome from damage during the freeze–thawing process. A greater degree of acrosomal normality had a positive influence on capacitation, highlighting the potential benefits of maintaining acrosomal integrity for sperm function (*p* < 0.05).

### 3.4. Mitochondrial Membrane Potential

**Figure 4:**
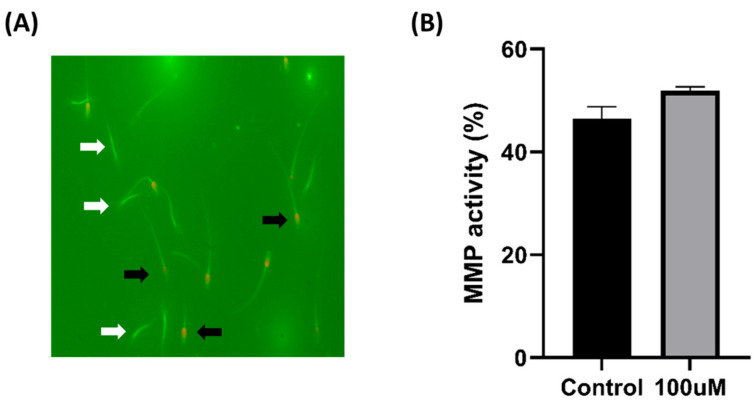
Effect of MnTBAP on pig sperm mitochondrial membrane potential during cryopreservation. (**A**) The white arrows point to living spermatozoa with high mitochondrial membrane potential (MMP), while the black arrows indicate dead spermatozoa that are weakly stained and have low MMP. (**B**) The percentage of sperm with high MMP. Data are indicated as means ± S.E.M.

The MMP was high and the spermatozoa were alive when the midsection was strongly fluorescent and the head was not stained with PI, as shown in Figure 4A. Regarding mitochondrial membrane integrity, the MnTBAP group demonstrated higher levels than those in the control group, although the difference between the two groups was not statistically significant (Figure 4B).

### 3.5. DNA Fragmentation

**Figure 5:**
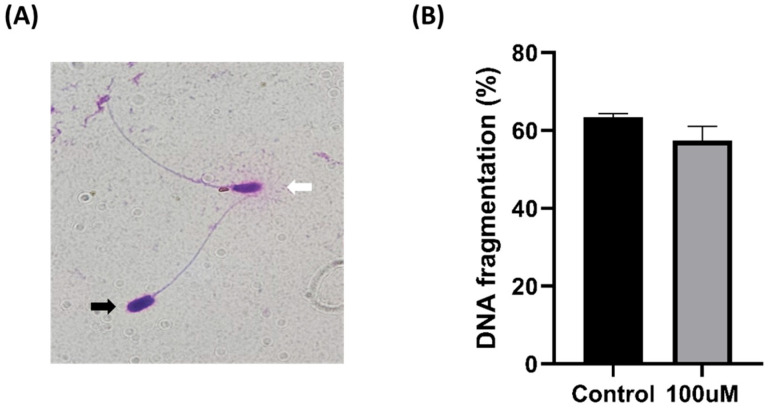
Effect of MnTBAP on pig sperm DNA fragmentation during cryopreservation. (**A**) The white arrow indicates a big halo around the sperm head and fragmented DNA, while the black arrow indicates a small or absent halo around the sperm head and non-fragmented DNA. (**B**) The ratio of DNA fragmented sperm from stained samples. Data are indicated as means ± S.E.M.

We investigated whether structural and physiological alterations occurring in sperm during freezing and thawing affect their genetic integrity. We assessed the degree to which damage to the sperm head and membrane resulted in DNA fragmentation. However, no significant differences were observed between the two experimental groups (Figure 5).

### 3.6. Caspase Activity

**Figure 6:**
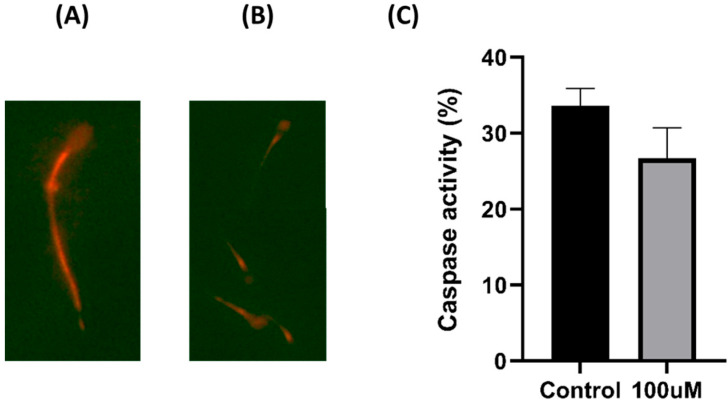
Effect of MnTBAP in the cryoprotectant solution on pig sperm’s caspase activity. (**A**) The strong staining of the midsection and tail shows a positive condition. (**B**) The weak staining indicates a negative condition. (**C**) The percentage of caspase activity. Data are indicated as means ± S.E.M.

In Figure 6, strong red light is observed in the midsection and tail of the sperm in (A) of the image, whereas (B) displays weaker fluorescence. We divided the positive and negative cells based on these illustrations. Caspase activity, an indicator of apoptosis, typically increases during apoptosis. Although the caspase activity tended to decrease in the treated group, no statistically significant differences were observed between the two experimental groups (Figure 6).

### 3.7. Plasma Membrane Lipid Disorder

**Figure 7:**
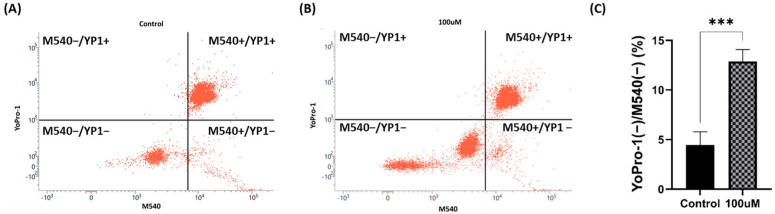
(**A**) The distribution of the control group in flow cytometry (merocyanine 540: M540 and YoPro-1: YP1). (**B**) The distribution of the 100 μM MnTBAP group in flow cytometry. (**C**) The ratio of the intact plasma membrane lipid arrangement to living cells. The four groups were divided as follows (1) M540+/YoPro-1+ and M540−/YoPro-1+: dead spermatozoa; (2) M540+/YoPro-1−: live spermatozoa with high lipid disorder; and (3) M540−/YoPro-1−: live spermatozoa with low lipid disorder. The asterisk indicates a significant difference between the two groups (*** *p* < 0.001). Error bars show the S.E.M.

The flow cytometry results in Figure 7 illustrate the plasma membrane lipid disorder of both the control group (A) and the 100 μM treatment group (B). In addition, Figure 7 (C) shows the percentage of viable sperm with a normalized lipid membrane arrangement, excluding deceased sperm. The treated group maintained more than twice the normalized membrane compared with that of the control group (*p* < 0.001).

### 3.8. Apoptosis Level

**Figure 8:**
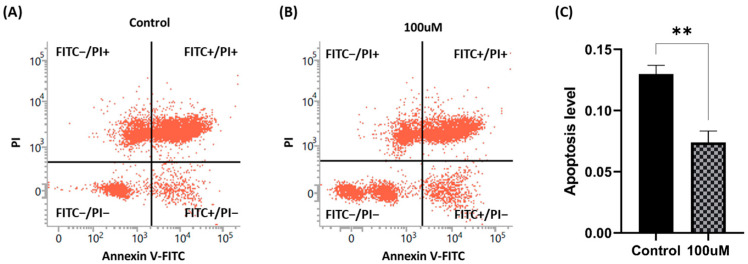
Effect of MnTBAP on apoptosis after the freezing–thawing process, showing the flow cytometry distribution aspects of (**A**) the non-treated group and (**B**) the 100 μM MnTBAP-treated group. (**C**) The apoptosis index. The four groups were divided as follows: (1) FITC−/PI−: viable and non-apoptotic; (2) FITC+/PI−: viable, phosphatidylserine (PS) translocated, and pro-apoptotic; (3) FITC+/PI+: dead and PS translocated; and (4) FITC−/PI+: late necrotic sperm (fluorescein isothiocyanate: FITC and propidium iodide: PI). The asterisk indicates a significant difference between the two groups (** *p* < 0.01). Data are indicated as means ± S.E.M.

The control flow cytometry result for the incidence of apoptosis is depicted in (A) of Figure 8, while (B) represents the result of the 100 μM treatment. The assessment of apoptosis incidence (C) in live sperm underscores the effectiveness of MnTBAP in reducing apoptosis (*p* < 0.01).

### 3.9. ROS Level

**Figure 9:**
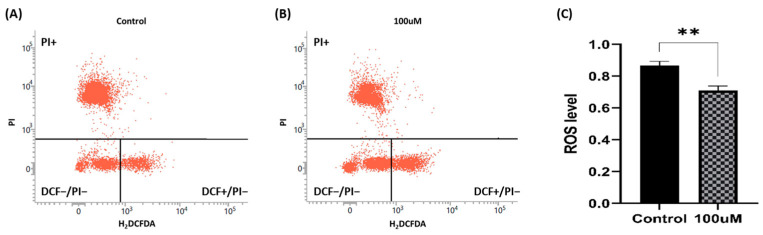
Effect of MnTBAP on ROS levels after the freezing–thawing process, showing the flow cytometry distribution aspects of (**A**) the non-treated group, and (**B**) the 100 μM MnTBAP-treated group. (**C**) The apoptosis index. The three groups were divided as follows (1) PI+: dead sperm; (2) DCF−/PI−: viable and low H_2_O_2_ level; and (3) DCF+/PI−: alive and high H_2_O_2_ level (2′,7′-dichlorodihydrofluoresceindiacetate: DCF and propidium iodide: PI). Data are indicated as means ± S.E.M. The asterisk indicates a significant difference between the two groups (** *p* < 0.01).

Distribution diagrams of ROS levels measured by flow cytometry are presented in Figure 9A,B. The graph in Figure 9C describes the H_2_O_2_ levels in live cells. The data indicate that the addition of 100 μM MnTBAP to the freezing media significantly suppresses the production of oxidative substances. Data are indicated as means ± S.E.M. (*p* < 0.01).

### 3.10. Gene Expression

**Figure 10:**
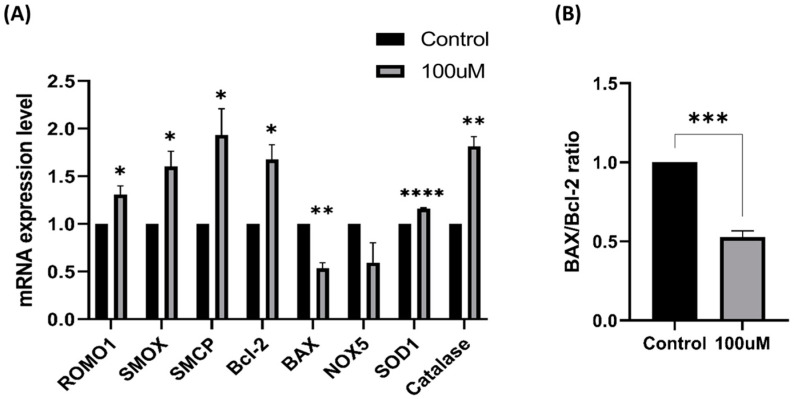
Effect of MnTBAP on gene expression related to the sperm status after freeze–thawing. (**A**) *ROMO1*: reactive oxygen species modulator 1; *SMOX*: spermine oxidase; *SMCP*: sperm mitochondria-associated cysteine-rich protein; *Bcl-2*: Bcl-2 apoptosis regulator; *BAX*: Bcl-2-associated X protein; *NOX5*: NADPH oxidase 5; *SOD1*: superoxide dismutase 1; and *catalase*. (**B**) Ratio of *BAX* and *Bcl-2*. The asterisk indicates a significant difference between the groups (* *p* < 0.05, ** *p* < 0.01, *** *p* < 0.01, and **** *p* < 0.0001). Error bars show the S.E.M.

In the treated group, upregulation of *ROMO1*, a regulator of oxidative stress; *SMOX*, which impacts regular development and maturation; and *SMCP*, which is involved in cell motility and structure formation, was observed. Additionally, the expression of *Bcl-2*, which is associated with anti-apoptosis, was upregulated, whereas that of *BAX*, which is related to apoptosis, was suppressed. This indicated the potential effectiveness of MnTBAP in protecting against apoptosis, as reflected by an approximately two-fold increase in the ratio between the expression levels of these two genes. Furthermore, *SOD1* and *catalase* levels, indicative of MnTBAP’s antioxidative effect, were notably escalated at a concentration of 100 μM MnTBAP (Figure 10).

### 3.11. Embryo Development

**Figure 11:**
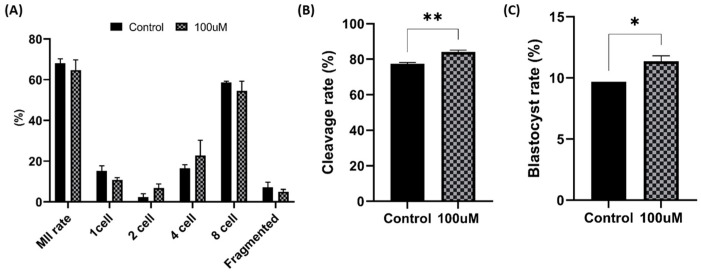
Effect of MnTBAP on embryo development after sperm cryopreservation. (**A**) Maturation rate (MII rate) and each developed stage (1-, 2-, 4-, and 8-cell stages and fragmented cells). (**B**) Cleavage rate on day 2. (**C**) Blastocyst rate on day 7. The asterisk indicates a difference between the groups. Error bars show the S.E.M. *: *p* < 0.05 and **: *p* < 0.01.

We standardized the metaphase II (MII) rate ratio by thawing both groups and adjusting the proportion of motile sperm based on the motility results of the CASA system. On day 2, the MnTBAP-treated group showed higher cleavage rates at the 2-, 4-, and 8-cell stages and lower tendencies for the 1-cell stage and fragmentation. Furthermore, blastocyst measurements on day 7 revealed a significantly higher rate. These findings suggested that the addition of MnTBAP to freezing media positively influenced the capacitation ability and embryo development (Figure 11).

## 4. Discussion

Excessive generation of ROS during sperm cryopreservation can result in impaired sperm motility and viability, morphological defects, and genetic aberrations, ultimately hindering fertilization or leading to cell death. The elevated concentration of unsaturated fatty acids in pigs makes them particularly vulnerable to the adverse effects of freezing, including cold shock, pH, alterations in the osmotic balance, and exposure to oxidative conditions [39,40,41]. In particular, ROS are major factors in the deleterious effects of various pathological conditions, including cell aging; dysfunction of DNA, proteins, and lipids; and apoptosis [42,43]. Hence, sperm quality may be enhanced by supplementing the cryopreservation medium with antioxidants, which can mitigate oxidative stress by attenuating ROS production. The aim of this study was to explore the effect of reduced ROS stress on both sperm structural integrity and capacitation competence by incorporating manganese (III) tetrakis (4-benzoic acid) porphyrin chloride (MnTBAP) into the cryoprotectant during the cryopreservation of swine semen.

MnTBAP is a synthetic antioxidant that exhibits cell permeability and possesses antioxidative properties comparable to those of superoxide dismutase (SOD). MnTBAP demonstrates pro-angiogenic abilities in cardiovascular diseases and has been utilized across various tissues and cells to enhance tolerance to oxidative stress conditions [23,44]. In horses, this chemical has been demonstrated to enhance motility, plasma membrane integrity, and the MMP, facilitate fertilization, and maintain lipid homeostasis [27]. Consistent with previous research, we observed elevated levels of motility, viability, and normal lipid organization compared with those of the control group in this study. ROS assault disrupts the structural integrity of the lipid matrix, leading to spontaneous peroxidation. However, treatment with 100 μM MnTBAP effectively ameliorated the generation of ROS, therefore potentially reducing membrane damage inflicted by ROS. In studies on freezing stallion sperm, 300 μM MnTBAP treatment resulted in decreased motility and compromising the ordered membrane compared with that of the control group, indicating that high concentrations of MnTBAP may exhibit cytotoxic effects [45]. In addition, the variations in the efficacy levels of MnTBAP observed across different species, such as in sheep, horses, and humans, are likely attributable to species-specific factors of dissimilarities in the methodology used in the experiments [29,46,47].

As the primary source of ROS, mitochondrial ROS (mtROS) are the key contributors to cellular damage. MnTBAP has been shown to mitigate mtROS production, thereby alleviating oxidative-stress-induced osteoblasts [48,49]. We evaluated MMP to ascertain whether this mechanism extends to porcine sperm and observed no significant variance between the untreated control and the 100 μM MnTBAP-treated group. This suggested that mtROS generation is contingent upon mitochondrial functionality, with the expression levels of *SMOX* and *SMCP* genes relatively elevated under the 100 μM treatment. *SMOX*, a gene closely linked to spermine activity and a polyamine abundant in mammalian seminal plasma, plays a crucial role in ion channel regulation, cell cycle modulation, defense against oxidative stress, preservation of membrane integrity, and cellular stability [50]. *SMOX* is implicated in polyamine biosynthesis through the catalysis of the spermine oxidation of spermidine, a process linked to hydrogen peroxide (H_2_O_2_) production. Polyamines play indispensable roles in cellular metabolism, division, and homeostasis [51]. They activate the mTOR cell signaling pathways, culminating in the synthesis of proteins crucial for sperm motility. Emerging studies have underscored their significance in processes such as blastocyst formation, implantation, and fetal growth [52]. Our findings demonstrated that the MnTBAP-treated group exhibited enhanced embryo development, blastocyst formation, and progressive motility, likely due to the upregulation of *SMOX*. Furthermore, the relatively high viability observed in the 100 μM treatment group suggested heightened metabolic activity in live spermatozoa, potentially inducing oxidative stress. The elevated *SMOX* expression indicates that MnTBAP treatment diminishes ROS levels formed during the freeze–thaw processes, consequently augmenting membrane lipid organization. In addition to the mitochondria, other cellular compartments, such as the cytoplasm and endoplasmic reticulum, serve as sources of ROS. Therefore, it is imperative to identify the specific ROS species targeted by MnTBAP and to investigate the underlying mechanisms involved [53].

The change in calcium dynamics during cryopreservation increases intracellular translocation and activation of the acrosome reaction (AR), leading to embryo development [46]. Freezing and thawing induce restructuring of the plasma membrane and initiate sperm capacitation. However, if excessively activated, capacitation can result in lipid disorders, thereby damaging the membrane composition [54]. Merocyanine 540, a probe known for its increased affinity for the plasma membrane as the lipid composition becomes disordered, was used to identify lipid disorders in this study. Bicarbonate and CO_2_ are recognized as capacitation effectors and induce rapid changes in the lipid architecture of porcine sperm plasma membranes. It is hypothesized that these components exert their effects early in the capacitation process, causing restructuring of the typically ordered membrane, thereby facilitating successful fertilization [55]. Under the experimental conditions of this study, higher cleavage and blastocyst rates were observed when fertilization was performed using spermatozoa treated with 100 μM MnTBAP. This is primarily attributed to the activation of the ROS defense mechanism, which effectively preserves sperm viability and acrosomal integrity, thereby enabling normal activated capacitation. Different concentrations of manganese (III) meso-tetrakis (N-ethylpyridinium-2-yl) porphyrin chloride (MnTE), a superoxide dismutase agent similar to MnTBAP, in the cryomedia enhanced embryo development in goats by protecting the sperm from ROS-induced DNA damage [28]. In contrast, MnTBAP in mice typically results in a decreased percentage of cleaved and hatched blastocysts compared with that of the untreated control. This discrepancy is likely attributable to variations in the composition and concentration of the extenders and capacitation media utilized for each species [29,47].

Metalloporphyrins, which feature redox-active transition metals bound to cyclic porphyrin core ligands, are important in reducing oxidative stress in biological systems. Through side-chain substitutions, these compounds can fine-tune their redox properties, serving as potent mimics of superoxide dismutase and regulators of transcription factor functions and demonstrating efficacy in treating various neurological disorders [56]. Metalloporphyrin SOD mimetics have been shown to inhibit neuronal apoptosis and promote the maintenance of live horse sperm by reducing ROS levels at low temperatures [57,58]. Similarly, our investigation revealed that treatment with MnTBAP led to the upregulation of genes associated with anti-apoptosis and the downregulation of apoptosis-related genes in boar sperm cryopreservation. Furthermore, the *BAX*/*Bcl-2* ratio between the two groups exhibited an approximate two-fold difference, indicating the antioxidative role of MnTBAP. This antioxidative effect is closely linked to the inhibition of apoptosis, ultimately resulting in enhanced cell viability.

MnTBAP has been utilized in semen cryopreservation among many species to explore its potential as a redox scavenger. However, comprehensive investigations at the protein level or within signaling pathways remain scarce. Although our study has demonstrated the beneficial impact of 100 μM MnTBAP on freezing outcomes, the blastocyst formation rate still falls below that of non-frozen semen or in vivo conditions. Therefore, further research is warranted to elucidate the underlying mechanisms and gain a deeper understanding of the effects of MnTBAP. Nonetheless, cryopreservation is a valuable assisted reproductive technology, and ongoing research efforts should aim to enhance its efficiency. In summary, our findings affirm that applying MnTBAP to porcine semen being frozen positively influences motility, viability, acrosome integrity, plasma membrane lipid composition, apoptosis, ROS levels, and embryo development.

## 5. Conclusions

In conclusion, we observed that adding 100 μM MnTBAP as a cryoprotectant had a positive effect by reducing ROS levels during the freeze–thaw process. This environment results in high viability and low apoptosis of spermatozoa, allowing them to preserve their membrane lipid arrangement and structural morphology, including that of the acrosome. The gene expression related to these parameters supports these findings. Cleavage and blastocyst rates were higher in the MnTBAP-treated group after fertilization, indicating that MnTBAP supported sustained cellular metabolism. Therefore, adding MnTBAP to porcine sperm cryopreservation induced an antioxidative effect, which positively contributed to the maintenance of sperm status. 

## Figures and Tables

**Table 1:** Primer sequences used for the gene expression analysis.

Gene	Primer Sequence (5′–3′)	Amplicon Length (bp)	Accession Number
*GAPDH*	F: ACTCACTCTTCTACCTTTGATGCT R: TGTTGCTGTAGCCAAATTCA	100	DQ_178124
*ROMO1*	F: GAAGATGGGCTTTGTGATGG R: ATAGTACATGGGCTGGGACT	401	NM_001097462.2
*SMOX*	F: TGGAAGAGACAACTGATGGG R: CATGGTTATGGTCACCCTCA	570	NM_001185170.1
*SMCP*	F: AGTGCACCTGCCTGAATAAG R: CCTACTTGTTTGGCTGCTTC	140	NM_001008685.1
*Bcl-2*	F: TGGTGGTTGACTTTCTCTCC R: ATTGATGGCACTAGGGGTTT	134	NM_214285.1
*BAX*	F: CAGCTCTGAGCAGATCATGA R: TTGAGACACTCGCTCAACTT	150	XM_003127290.5
*NOX5*	F: TTCTTCGCCCTCTTTGACTT R: CAGTCAAAGTTGAGGCACTG	566	XM_021100544.1
*SOD1*	F: GCCAAAGGATCAAGAGAGGC R: TACACCACAGGCCAAACGAC	226	NM_001190422.1
*Catalase*	F: CGAAGGCGAAGGTGTTTG R: AGTGTGCGATCCATATCC	374	NM_214301.2

F: forward; R: reverse; *ROMO1*: reactive oxygen species modulator 1; *SMOX*: spermine oxidase; *SMCP*: sperm mitochondria-associated cysteine-rich protein; *Bcl-2*: Bcl-2 apoptosis regulator; *BAX*: Bcl-2-associated X protein; *NOX5*: NADPH oxidase 5; *SOD1*: superoxide dismutase 1, *Catalase*.

## Data Availability

The data described in this study are available upon request from the corresponding author.
